# Improving the Quality and Completeness of Clinical Handover Using the Situation, Background, Assessment, Recommendation (SBAR) Framework: A Closed-Loop Quality Improvement Study at Almanagil Teaching Hospital, Sudan

**DOI:** 10.7759/cureus.109293

**Published:** 2026-05-20

**Authors:** Mohamed Mobark Obed Yousif, Mayamen Mugahid Mohamed Abdelgalil, Saria Elsiddig Mohamed Ali, Hussein Malik Khidir Hamad, Rawan Mohamedahmed Khider Ali, Areeg Elnag Hamed Elshiekh Abdelbagi, Esraa Ismat Abdulrahim Abdulaal, Yousif Abdalla Yousif Ahmed, Duaa Elnour Gadelseed Elnour, Mustafa Mohamed

**Affiliations:** 1 Faculty of Medicine, Bayan University, Khartoum, SDN; 2 Department of Emergency Medicine, Al-Shamal Specialized Hospital, Dongola, SDN; 3 Department of General Medicine, Al-Shamal Specialized Hospital, Dongola, SDN; 4 Department of Emergency Medicine, Almanagil Teaching Hospital, Managil, SDN; 5 Department of Emergency Medicine, University of Gezira, Wad Madani, SDN; 6 Department of Internal Medicine, AL Neelain University, Khartoum, SDN

**Keywords:** audit, clinical handover, communication, documentation, patient safety, quality improvement, sbar, sudan

## Abstract

Background

Effective communication during clinical handover is essential for patient safety, yet deficiencies in structured communication remain common, particularly in resource-limited settings. The Situation, Background, Assessment, Recommendation (SBAR) framework has been widely recommended to standardize communication and reduce errors.

Methods

A closed-loop quality improvement project was conducted at Almanagil Teaching Hospital, Managil, Gezira State, Sudan, using a two-cycle audit design structured around the Plan-Do-Study-Act (PDSA) framework. Compliance with SBAR communication standards was assessed among doctors involved in clinical handover. Data were collected from 48 handover events in cycle 1 (baseline) and 44 events in cycle 2 (post-intervention). A multifaceted intervention, including structured education, standardized SBAR templates, visual reminders, and continuous feedback, was implemented between cycles. Data were analyzed using descriptive statistics and the chi-square test, with a p-value of <0.05 considered statistically significant.

Results

A total of 92 handover events were analyzed. Significant improvements were observed across most SBAR components following the intervention. Documentation of key elements such as patient identifiers, clinical status, diagnosis, and vital signs increased markedly, with several parameters reaching near-complete compliance (p < 0.001). Critical communication elements, including escalation plans, actions, and response to treatment, also demonstrated substantial improvement. However, some variables, including documentation of treatment given, patient location, and gender, remained below the target compliance threshold.

Conclusion

The implementation of a structured SBAR-based quality improvement intervention significantly enhanced the completeness and quality of clinical handover documentation. These findings support the use of simple, low-cost, and scalable interventions to improve communication practices and patient safety, particularly in resource-constrained settings. Ongoing monitoring and reinforcement are required to sustain improvements and address remaining gaps.

## Introduction

Effective communication among healthcare professionals is a fundamental component of patient safety and high-quality clinical care. Failures in communication, particularly during patient handover and clinical escalation, have been consistently identified as a major contributor to adverse events, including medication errors, delayed treatment, and preventable morbidity and mortality [1,2].

Clinical handover represents a critical point in patient care where responsibility is transferred between healthcare providers. During this transition, incomplete or unstructured communication can lead to the loss of essential clinical information, duplication of work, or inappropriate clinical decisions. Studies have demonstrated that communication breakdowns during handover are among the most common causes of preventable patient harm in hospital settings [2].

To address these challenges, structured communication tools have been introduced to standardize information exchange and reduce variability in clinical practice. One of the most widely adopted frameworks is the Situation, Background, Assessment, Recommendation (SBAR) tool, which provides a clear and systematic method for conveying critical clinical information. SBAR has been endorsed by major healthcare organizations, including the World Health Organization and the Joint Commission, as an effective strategy to enhance communication and promote patient safety [1].

Evidence from systematic reviews and interventional studies suggests that the implementation of SBAR improves the clarity, completeness, and efficiency of communication among healthcare professionals. It has been associated with improved teamwork, reduction in communication-related errors, and enhanced patient safety outcomes [3,4].

Furthermore, structured handover tools such as SBAR have been shown to increase the accuracy of information transfer and reduce the incidence of clinical errors, particularly in high-risk environments such as emergency departments and intensive care units. The use of standardized communication frameworks ensures that critical elements of patient information are consistently communicated, thereby minimizing omissions and enhancing continuity of care [5].

Despite the well-documented benefits of SBAR, compliance with its use remains inconsistent in many healthcare settings, particularly in resource-limited environments. Studies have shown that healthcare professionals often fail to utilize all components of SBAR effectively, with incomplete documentation and variability in communication practices persisting even after training interventions [6].

In low- and middle-income countries, including Sudan, the challenges are further compounded by high patient volumes, limited staffing, and the absence of standardized communication protocols. These factors contribute to variability in clinical handover practices and increase the risk of communication-related errors. However, there remains a paucity of local data evaluating the implementation and effectiveness of structured communication tools such as SBAR in these settings [7].

Therefore, this study aimed to assess baseline compliance with SBAR communication standards among doctors at Almanagil Teaching Hospital, Managil, Gezira State, Sudan, identify gaps in practice, and evaluate the impact of a structured quality improvement intervention on enhancing the completeness and compliance of SBAR handover documentation.

## Materials and methods

Study design

**Figure 1:**
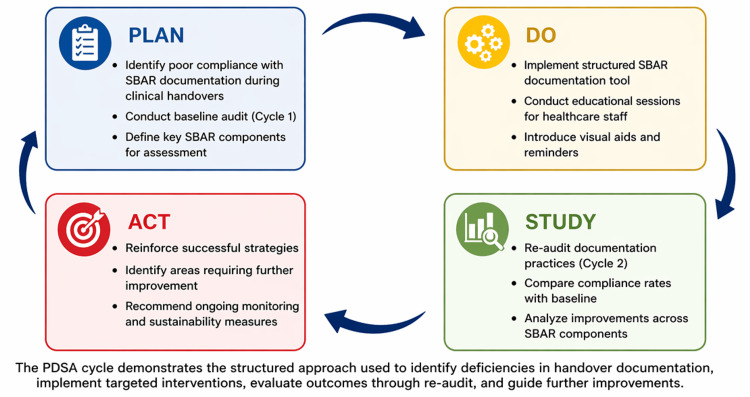
Plan–Do–Study–Act (PDSA) cycle illustrating the quality improvement framework for Situation, Background, Assessment, Recommendation (SBAR) documentation The PDSA cycle demonstrates the structured quality improvement approach used in this study. In the planning phase, deficiencies in SBAR documentation during clinical handovers were identified and baseline data were collected. During the implementation phase, a structured SBAR documentation tool was introduced alongside staff education and visual reminders. The study phase involved re-audit of documentation practices and comparison with baseline findings. In the action phase, improvements were reinforced, remaining gaps were addressed, and strategies for sustainability were implemented to ensure ongoing enhancement of documentation quality. Created by the authors using Canva (Canva Pty Ltd., Sydney, Australia).

This study was conducted as a closed-loop quality improvement project using a two-cycle audit design to assess and improve compliance with SBAR communication standards among doctors. The project was structured in accordance with the Plan-Do-Study-Act (PDSA) framework, enabling iterative evaluation and targeted intervention to enhance clinical communication practices (Figure 1) [8].

Study setting

The study was carried out at Almanagil Teaching Hospital, a tertiary healthcare facility located in Managil, Gezira State, Sudan. The hospital provides emergency, inpatient, and outpatient services and serves a large population from both urban and rural areas. Clinical communication and patient handover are routinely conducted by doctors across different departments within the hospital.

Study population and sample size

The study population consisted of doctors involved in patient care and clinical handover at Almanagil Teaching Hospital. Data were collected over two audit cycles, comprising 48 handover/documentation events in the baseline phase (cycle 1) and 44 events in the post-intervention phase (cycle 2). All eligible handover/documentation events occurring during the predefined audit periods were included using a total coverage approach. Standardized audit criteria and a consistent methodology were applied across both audit cycles. 

The baseline audit (cycle 1) was conducted from January to February 2025. This was followed by a planning phase in March 2025 and an intervention period between April and May 2025. The re-audit (cycle 2) was conducted in June 2025, followed by data analysis in July 2025 and implementation of sustainability measures from August 2025 onward.

Audit standards

The audit assessed compliance with SBAR communication standards, encompassing the essential components of situation, background, assessment, and recommendation. A target compliance rate of ≥80% was defined as an achievable and clinically meaningful benchmark, consistent with quality improvement principles and reflecting the practical challenges of implementing structured communication in routine clinical settings.

Data collection

Data were collected using a pre-designed structured data collection tool based on SBAR components. The tool was developed to assess the completeness and quality of clinical communication and documentation.

The SBAR data collection tool consisted of four core domains: Situation, Background, Assessment, and Recommendation. Each component was assessed as a binary variable (1 = documented, 0 = not documented) based on predefined criteria. A situation was considered documented if the current clinical issue or reason for handover was clearly stated. Background was recorded if relevant clinical history, diagnosis, or clinical context was included. Assessment was considered present if the clinician’s evaluation or interpretation of the patient’s condition was documented. The recommendation was documented if a clear management plan, instruction, or required action was specified. Overall compliance was calculated as the proportion of documented components per handover event.

Data collection was performed by trained personnel to ensure consistency and reliability. Prior to data collection, the data collectors were oriented on the use of the tool to minimize interobserver variability. All collected data were reviewed for completeness and accuracy before analysis.

The unit of analysis in this study was a single handover/documentation event, defined as one patient-specific handover record reviewed during a clinical handover encounter. Each event corresponded to the documentation of an individual patient at the time of handover and was evaluated independently for compliance with SBAR components using the standardized data collection tool.

Intervention

Following the initial audit cycle, a multifaceted intervention was implemented to address the identified gaps in SBAR compliance. This included structured educational sessions focusing on SBAR communication principles, alongside the introduction of standardized SBAR documentation templates to facilitate consistent practice. In addition, visual reminders and educational posters were placed in key clinical areas to reinforce adherence to the SBAR framework. Continuous feedback was also provided to healthcare staff based on audit findings to promote engagement and sustain improvement.

The educational intervention was delivered over a three-week period, during which structured training sessions were conducted twice weekly. Each session lasted approximately 30-45 minutes and targeted doctors involved in clinical handover. The sessions included interactive teaching on SBAR principles, practical examples, and case-based discussions to reinforce application in routine clinical practice. Collectively, these interventions were designed to enhance awareness, standardize communication practices, and promote sustained behavioral change among clinicians.

Re-audit (cycle 2)

**Figure 2:**
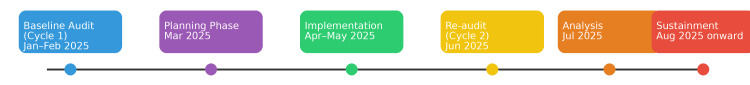
Intervention timeline of the quality improvement project (2025) The timeline illustrates the sequence of the quality improvement process conducted in 2025, including baseline data collection (cycle 1), intervention planning and implementation, post-intervention re-audit (cycle 2), and subsequent analysis and sustainability planning. The structured progression of these phases ensured consistency in methodology and enabled reliable evaluation of the intervention’s impact on Situation, Background, Assessment, Recommendation (SBAR) documentation practices. Created by the authors using Canva (Canva Pty Ltd., Sydney, Australia).

A second audit cycle was conducted following the implementation of the intervention to evaluate its effectiveness in improving compliance with SBAR communication standards. The re-audit was performed over a comparable time frame using the same standardized data collection tool and methodology to ensure consistency and comparability between cycles. This methodological approach minimized potential measurement bias and allowed for a reliable assessment of the impact of the intervention on clinical communication practices. The sequence of audit cycles, intervention implementation, and evaluation phases is illustrated in the intervention timeline (Figure 2).

Data analysis

Data were entered and cleaned using Microsoft Excel (Microsoft Corporation, Redmond, WA, USA) and analyzed using JASP (Version X.X; JASP Team). Descriptive statistics were used to summarize compliance with individual SBAR components in each audit cycle, and results are presented as frequencies and percentages (n, %).

For each documentation variable, compliance between the pre-intervention (cycle 1) and post-intervention (cycle 2) phases was compared as a binary outcome (documented vs not documented) using 2 × 2 contingency tables. Pearson’s chi-square test was applied when its assumptions were satisfied, while Fisher’s exact test was used for variables with small expected cell counts.

To quantify the magnitude of change, absolute differences in proportions between cycles were calculated. Additionally, Cramér’s V was reported as a measure of effect size. All statistical tests were two-tailed, and a p-value of <0.05 was considered statistically significant.

Ethical considerations

This study was conducted as a quality improvement initiative and did not involve direct patient intervention. All data were anonymized, and no identifiable patient information was collected. The study adhered to institutional policies and ethical principles in accordance with the Declaration of Helsinki.

## Results

A total of 92 handover/documentation events were analyzed across two audit cycles, including 48 events in the baseline phase (cycle 1) and 44 events following the intervention (cycle 2). Overall, there was a substantial improvement in compliance with SBAR documentation standards across most assessed parameters following implementation of the quality improvement intervention.

Documentation of core patient identifiers improved markedly, with large effect sizes observed. Recording of patient name increased from 14 (29.2%) in cycle 1 to 44 (100%) in cycle 2 (p < 0.001), while hospital name and date improved from 8 (16.7%) to 44 (100%) (p < 0.001 for both). Documentation of department name, time, and handover details (handover from and handover to), which were absent in cycle 1, reached high levels of compliance in cycle 2 (84.1%-100%, all p < 0.001), reflecting substantial improvements.

Significant improvements were also observed in clinical information components, with moderate to large effect sizes across variables. Documentation of reason for admission increased from 16 (33.3%) to 44 (100%) (p < 0.001), while urgent issues improved from two (4.2%) to 44 (100%) (p < 0.001). Recording of current diagnosis, laboratory/imaging findings, and clinical status demonstrated substantial gains, rising from 20.8%, 2.1%, and 31.3% in cycle 1 to 97.7%, 86.4%, and 90.9% in cycle 2, respectively (all p < 0.001). Documentation of vital signs also improved from 8 (16.7%) to 38 (86.4%) (p < 0.001).

Assessment and recommendation components of SBAR showed consistent and significant enhancement, with predominantly large effect sizes. Documentation of impression and response to treatment increased from 6.3% and 2.1% in cycle 1 to 77.3% and 79.5% in cycle 2, respectively (p < 0.001 for both). Similarly, recommendation-related elements. including actions, escalation plans, pending investigations, and review timelines, demonstrated marked improvements, with escalation plans increasing from 0% to 97.7% and actions from 6.3% to 95.5% (p < 0.001).

Despite these overall improvements, some variables demonstrated smaller effect sizes and remained suboptimal in the post-intervention phase. Documentation of treatment given increased from one (2.1%) to eight (18.2%) (p = 0.009). Improvements in gender (27.1% vs 47.7%, p = 0.042) and location/bed documentation (14.6% vs 47.7%, p = 0.001) were observed but did not reach high compliance levels.

Additional gains were noted in safety and continuity-of-care elements. Documentation of allergies/past medical history improved from 29.2% to 61.4% (p = 0.002), while red flags increased from 0% to 61.4% (p < 0.001) and instructions from 25.0% to 70.5% (p < 0.001). Documentation of professional accountability also improved significantly, with handover physician signatures increasing from 27.1% to 93.2% and receiving physician signatures from 2.1% to 88.6% (p < 0.001 for both), reflecting large effect sizes.

Overall, statistically significant improvements were observed across nearly all SBAR components, with most variables demonstrating substantial increases in compliance and moderate to large effect sizes following the intervention (Table 1).

**Table 1:** Comparison of compliance with Situation, Background, Assessment, Recommendation (SBAR) documentation components between baseline (cycle 1) and post-intervention (cycle 2) phases Values are presented as frequencies and percentages (n, %). Absolute differences represent percentage point changes between audit cycles. Comparisons were performed using 2 × 2 contingency tables with Pearson’s chi-square test, and Fisher’s exact test was applied where appropriate due to small expected cell counts. Cramér’s V was calculated as a measure of effect size. A p-value of <0.05 was considered statistically significant.

Parameter	Cycle 1 n (%)	Cycle 2 n (%)	Absolute difference (% points)	χ² (df=1)	Cramér’s V	p-value
Patient name	14 (29.2%)	44 (100.0%)	70.8	49.44	0.733	<0.001
Hospital name	8 (16.7%)	44 (100.0%)	83.3	64.87	0.84	<0.001
Department name	0 (0.0%)	37 (84.1%)	84.1	67.52	0.857	<0.001
Date	8 (16.7%)	44 (100.0%)	83.3	64.87	0.84	<0.001
Time	0 (0.0%)	44 (100.0%)	100	92	1	<0.001
Handover from	0 (0.0%)	44 (100.0%)	100	92	1	<0.001
Handover to	0 (0.0%)	44 (100.0%)	100	92	1	<0.001
Location/bed	7 (14.6%)	21 (47.7%)	33.1	11.54	0.354	0.001
Age	14 (29.2%)	44 (100.0%)	70.8	49.44	0.733	<0.001
Gender	13 (27.1%)	21 (47.7%)	20.6	4.12	0.212	0.042
Reason for admission	16 (33.3%)	44 (100.0%)	66.7	43.32	0.686	<0.001
Urgent issues	2 (4.2%)	44 (100.0%)	95.8	81.06	0.939	<0.001
Relevant medications	12 (25.0%)	34 (77.3%)	52.3	25.29	0.524	<0.001
Current diagnosis	10 (20.8%)	43 (97.7%)	76.9	58.39	0.797	<0.001
Lab/images	1 (2.1%)	38 (86.4%)	84.3	65.85	0.846	<0.001
Treatment given*	1 (2.1%)	8 (18.2%)	16.1	6.74	0.271	0.009
Allergies/Past medical history	14 (29.2%)	27 (61.4%)	32.2	9.58	0.323	0.002
Clinical status	15 (31.3%)	40 (90.9%)	59.6	35.82	0.624	<0.001
Vital signs	8 (16.7%)	38 (86.4%)	69.7	45.04	0.7	<0.001
Impression	3 (6.3%)	34 (77.3%)	71	52.45	0.755	<0.001
Response to treatment	1 (2.1%)	35 (79.5%)	77.4	57.07	0.788	<0.001
Red flags	0 (0.0%)	27 (61.4%)	61.4	39.28	0.653	<0.001
Actions	3 (6.3%)	42 (95.5%)	89.2	76.2	0.91	<0.001
Pending investigations	2 (4.2%)	30 (68.2%)	64	40.44	0.663	<0.001
Escalation plan	0 (0.0%)	43 (97.7%)	97.7	87.91	0.978	<0.001
Review timeline	0 (0.0%)	30 (68.2%)	68.2	45.88	0.706	<0.001
Instructions	12 (25.0%)	31 (70.5%)	45.5	19.04	0.455	<0.001
Handover physician signature	13 (27.1%)	41 (93.2%)	66.1	42.76	0.682	<0.001
Receiving physician signature	1 (2.1%)	39 (88.6%)	86.5	70.73	0.877	<0.001

## Discussion

The findings of this study demonstrate a substantial improvement in compliance with SBAR documentation standards following the implementation of a structured, multifaceted quality improvement intervention. Across nearly all assessed parameters, documentation completeness improved significantly, indicating that targeted interventions can effectively address deficiencies in clinical communication and handover practices.

Effective communication during clinical handover is widely recognized as a cornerstone of patient safety. Communication failures have been consistently identified as a major contributor to adverse events, particularly during transitions of care [9]. Structured communication tools have therefore been widely promoted to reduce variability and enhance information transfer. The SBAR framework, in particular, has been extensively adopted as a standardized method to improve communication clarity and patient safety [10]. Furthermore, systematic evidence suggests that SBAR implementation is associated with improvements in patient safety outcomes, although the strength of evidence varies across settings [3].

The introduction of SBAR-based interventions in this study resulted in significant improvements in both administrative and clinical documentation components. Improvements in core identifiers, clinical status, diagnosis, and vital signs reflect enhanced completeness and clarity of information transfer. These findings are consistent with previous studies demonstrating that structured communication frameworks improve documentation accuracy, reduce omissions, and enhance the reliability of clinical handovers [4]. Additionally, quality improvement studies implementing SBAR have shown that combining education, standardized tools, and reinforcement strategies leads to measurable improvements in communication practices [11].

Notably, the most pronounced improvements were observed in previously under-documented domains, such as urgent issues, escalation plans, and response to treatment. These elements are critical for timely clinical decision-making and patient safety, particularly in high-acuity settings. The substantial increase in compliance in these domains suggests that the intervention not only improved documentation practices but also enhanced clinicians’ awareness of structured communication principles. This aligns with evidence indicating that SBAR-based training programs significantly improve communication effectiveness and patient safety culture [12].

Despite these improvements, certain variables remained suboptimal in the post-intervention phase, including documentation of treatment given, patient location, and gender. Similar findings have been reported in previous quality improvement studies, where some aspects of documentation demonstrate slower improvement due to variability in clinician engagement or perceived importance of specific data elements [11]. These residual gaps highlight the need for continuous reinforcement, ongoing audit cycles, and potentially integration of structured templates into routine clinical workflows.

The findings of this study are particularly relevant in the context of low- and middle-income healthcare settings, where challenges such as high patient loads, limited staffing, and lack of standardized communication systems are common. In such environments, communication failures remain a significant contributor to adverse events, emphasizing the importance of structured approaches like SBAR [9]. The successful implementation of a low-cost, multifaceted intervention in this study demonstrates the feasibility and scalability of similar strategies in resource-constrained settings. In addition, sustained improvement in structured communication practices may depend on strong ‎leadership engagement and a supportive organizational culture that encourages adherence to ‎evidence-based quality improvement initiatives and standardized clinical workflows [13].

From a broader perspective, this study reinforces the critical role of structured communication in improving patient safety, continuity of care, and clinical accountability. Enhanced documentation of escalation plans, red flags, and follow-up instructions is particularly important in reducing delays in care and ensuring appropriate clinical responses. Moreover, improvements in physician signatures reflect increased professional accountability, which is a key component of high-quality healthcare delivery.

This study has several strengths. It utilized a closed-loop audit design, allowing direct evaluation of intervention effectiveness. The use of a standardized data collection tool ensured consistency across audit cycles, and the comprehensive assessment of multiple SBAR components provided a detailed evaluation of communication practices. Additionally, the multifaceted nature of the intervention likely contributed to the magnitude of improvement observed.

However, several limitations should be acknowledged. First, the study was conducted in a single center, which may limit generalizability. Second, the relatively small sample size may affect statistical power for some variables. Third, the study relied on documentation as a proxy for communication quality and did not directly measure clinical outcomes such as adverse events. In addition, the absence of a concurrent control group limits the ability to attribute the observed improvements solely to the intervention, as temporal or contextual factors may have contributed to the changes. Furthermore, the assessment of multiple variables may increase the risk of type I error, and some variables had low baseline frequencies, which may affect the robustness of statistical comparisons. Future studies may also evaluate structured quality improvement methodologies, including Lean Six ‎Sigma-based approaches, which have been linked to enhanced patient safety, workflow ‎standardization, and operational efficiency across healthcare settings [14].

## Conclusions

The implementation of a structured, multifaceted quality improvement intervention was associated with a significant improvement in compliance with SBAR communication standards across most assessed parameters. These findings highlight the effectiveness of combining education, standardized documentation tools, and continuous feedback in enhancing the completeness and quality of clinical handover practices.

Despite these improvements, variability in compliance across certain components indicates the need for ongoing reinforcement, continuous audit cycles, and integration of structured communication tools into routine clinical workflows. Overall, this study demonstrates that simple, low-cost, and context-appropriate interventions can substantially improve communication practices, thereby supporting enhanced patient safety, continuity of care, and clinical accountability, particularly in resource-limited settings.
